# Effect of Non-Conventional Drying Methods on In Vitro Starch Digestibility Assessment of Cooked Potato Genotypes

**DOI:** 10.3390/foods8090382

**Published:** 2019-09-02

**Authors:** Christina E. Larder, Vahid Baeghbali, Celeste Pilon, Michèle M. Iskandar, Danielle J. Donnelly, Sebastian Pacheco, Stephane Godbout, Michael O. Ngadi, Stan Kubow

**Affiliations:** 1 School of Human Nutrition, McGill University, 21,111 Lakeshore, Ste. Anne de Bellevue, QC H9X 3V9, Canada (C.E.L.) (C.P.) (M.M.I.); 2 Bioresource Engineering, McGill University, 21,111 Lakeshore, Ste. Anne de Bellevue, QC H9X 3V9, Canada (V.B.) (M.O.N.); 3 Plant Science Department, McGill University, 21,111 Lakeshore, Ste. Anne de Bellevue, QC H9X 3V9, Canada; 4 Faculty of Engineering, Institut de recherche et de développement en agroenvironnement (IRDA), 2700, rue Einstein, Québec, QC G1P 3W8, Canada (S.P.) (S.G.); 5 Soil and Agricultural Engineering Department, Laval University, 2425 rue de l’Agriculture, Québec, QC G1V 0A6, Canada

**Keywords:** *Solanum tuberosum* L., starch, digestibility, freeze-drying, microwave vacuum drying, conductive hydro-drying, instant controlled pressure drop, processing

## Abstract

Potatoes (*Solanum tuberosum* L.) are a good dietary source of carbohydrates in the form of digestible starch (DS) and resistant starch (RS). As increased RS content consumption can be associated with decreased chronic disease risk, breeding efforts have focused on identifying potato varieties with higher RS content, which requires high-throughput analysis of starch profiles. For this purpose, freeze drying of potatoes has been used but this approach leads to inaccurate RS values. The present study objective was to assess the starch content (RS, DS and total starch (TS)) of three cooked potato genotypes that were dried using freeze drying and innovative drying techniques (microwave vacuum drying, instant controlled pressure drop drying and conductive hydro-drying) relative to freshly cooked potato samples. Depending on the genotype, all drying methods showed one or more starch measures that were significantly different from freshly cooked values. The combination of ultrasound and infrared assisted conductive hydro-drying was the only method identified to be associated with accurate assessment of DS and TS content relative to fresh samples. The drying treatments were all generally associated with highly variable RS content relative to fresh controls. We conclude that freshly cooked samples must be used for selecting varieties with a high proportion of RS starch as drying of cooked potatoes leads to unreliable RS measurements.

## 1. Introduction

Potatoes (*Solanum tuberosum* L.) are an important worldwide staple food crop, which serves as a good dietary source of carbohydrates, vitamin C, several B vitamins, antioxidants, minerals and protein [1,2,3,4,5,6,7]. The major carbohydrate component in potatoes is starch, which ranges from 70 to 90% on a dry mass basis, depending on genotype and environmental factors such as growing conditions [8,9,10]. Potato starch in its raw form is inedible but is digestible by humans when cooked [11]. Potato starch is composed of amylose and amylopectin, which are digested at different rates. Amylose is a linear polysaccharide molecule with glucose units linked by α1-4 bonds whereas amylopectin has both α1-4 and α1-6 bonds, which form branches that diverge from the main section [12]. The process of gelatinization occurs when the hydrogen bonds between amylose and amylopectin are broken following application of sufficient heat and water, which disrupts the starch granule [11]. During the above process, water molecules become bonded to the exposed hydroxyl groups of amylose and amylopectin. These new bonds lead to the swelling of the starch granules due to water uptake. The resulting disruption of starch grains and their starch structures leads to increased starch solubility. When there is a cooling period, starch molecules reassociate slowly but not with the same pre-heating level of organization, in a process called retrogradation [12,13,14]. Retrograded starch is generally more resistant to digestion with faster retrogradation occurring with amylose, due to its lack of branches as compared to amylopectin. The degree to which starch is digested determines the rate and extent of glucose release into the blood stream and can be calculated as glycemic index (GI) [11]. As intake of high GI foods has been associated with an increased risk of type-2 diabetes, cardiovascular disease and obesity [11,14], research has focused on identification of potato genotypes with relatively low starch digestibility [8]. Assessment of potato starch digestibility solely by measurement of amylose and amylopectin content is not sufficient as previous work has shown that starch digestibility and proportions of either amylose or total starch content were not correlated in cooked potatoes [2]. There are a variety of other intrinsic factors that can determine the rate of digestion of starch such as the degree of starch phosphorylation [15].

Another approach to assess the GI of potatoes is to classify the starch in terms of its degree of digestibility based on digestible starch (DS) and resistant starch (RS) content [8]. The DS component is composed of both rapidly digestible starch (RDS) and slowly digestible starch (SDS). As the names suggest, RDS is digested first. SDS is also completely digested in the small intestine although more slowly for reasons not yet fully understood [16]. RS can be defined as the starch portion that cannot be digested by enzymes in the small intestine and so reaches the colonic regions where it is fermented by colonic microbiota [17]. For this reason, it is classified as an insoluble dietary fiber [8,18]. The proportion of RS directly affects the glycemic impact of potatoes [19]. A greater dietary intake of RS is also associated with decreased risk of non-communicable diseases such as obesity and cardiovascular diseases [4,8,20]. Due to the high consumption of potatoes worldwide, selecting potato genotypes with greater RS concentrations concurrent with lower DS content can result in a relatively large impact on human health [3]. Towards this goal, it is important to standardize high-throughput methods used to process, prepare and analyze the starch quality of potatoes to support breeding efforts aimed at improving the starch quality of potato table stock.

Freeze-drying (FD) has commonly been used prior to starch quality assessment for both research and industry [21]. Lyophilized potato samples must be adjusted for their original tuber moisture content by calculating starch on a dry mass basis as moisture is a confounding variable for glucose release measurements [22]. Samples lyophilized by FD are dried to completion, before they are stored at −80 °C. A major concern regarding starch quality measurements is that FD of raw [21] or cooked [23] potato either overestimates or underestimates measurements of starch digestibility as compared to fresh potato samples. Previous work by our group and others has demonstrated that FD caused significant cracks and fragmentation of the starch granule cell wall integrity [23,24], which affects the permeability of the dried cooked potato starch to enzymatic digestion [23]. The change in starch digestibility occurs regardless whether FD is used on previously cooked potato tubers or if raw potatoes are FD, rehydrated and then cooked prior to analysis [23]. The effect of FD on starch estimates of RS, DS or total starch (TS) was shown to be genotype dependent [23]. RS values were either greatly underestimated or overestimated relative to freshly cooked potatoes depending on genotype after FD [23]. Consequently, FD was considered to lead to inaccuracies in estimating RS for genotype screening purposes. Differences in starch granule composition such as amylose content as well as starch granule surface area and size between varieties have been shown [25,26]. Furthermore, differences among amylose/amylopectin ratios, starch gelatinization properties, RS content and GI were observed between modified potatoes and mother line controls [18]. Therefore, genotypic differences in starch content and granule size in terms of sensitivity to drying could contribute to previously observed variability in starch estimates between varieties after drying [23].

Alternative drying technologies to FD are required for high throughput sample processing of cooked potatoes for accurate determination of starch quality. In that regard, recent novel food drying technologies have been developed as an alternative to FD, that are equivalent or superior in terms of preservation of heat-sensitive nutritional components. These include instant controlled pressure drop drying (Déshydratation par Détentes Successives in French, DDS), microwave vacuum drying (MVD) and conductive hydro-drying (CHD). The DDS process involves subjecting a sample to multiple rounds of pressure-drops until a desired moisture content is achieved [27]. Swell drying combines conventional hot air drying and DDS to reduce drying time and allow for a more efficient drying process, while retaining product quality [28,29]. MVD involves direct heating by microwaves emitted onto a sample coupled with a low-pressure environment created by a vacuum [30,31]. The vacuum creates a pressure gradient that favors a rapid migration of the vapor to the outside of the product, allowing for rapid drying and a decreased use of heat during the drying process [32]. A comparison between drying methods demonstrated that MVD outperformed FD for the retention of physico-chemical properties related to polyphenols, antioxidant capacity and physical parameters such as color and texture of dried food products [31]. CHD, also known as Refractance Window Drying (RWD), involves the spreading of moist samples over a semi-transparent Mylar plastic sheet that rests on a hot water bath typically set to 90–95 °C. The system uses hot water to transmit thermal energy to the material being dried in an efficient manner, both in regard to energy consumption and drying uniformity compared to other drying methods such as FD [33,34]. Samples using CHD are dried to completion. The term RWD was based on a presumed heat transfer mechanism that was proven to be negligible. Thus, the term CHD is more scientifically accurate to describe this technology.

The present study involved an investigation of the capacity of the above innovative drying processes to contribute to accurate assessment of starch digestibility of dried cooked potatoes in comparison to digestibility measures from freshly cooked potato samples. The tested drying methods included FD, DDS, MVD and CHD. Three well-characterized potato genotypes were subjected to in vitro starch digestion to assess for the DS, RS and TS content of the cooked potato samples after undergoing various drying treatments. Fluorescence microscopy and scanning electron microscopy (SM) were carried out to visually assess the effects of the drying methods on starch granule integrity.

## 2. Materials and Methods

### 2.1. Source Material

Organically grown potato tubers from three genotypes were obtained from the Bon Accord Seed farm operated by Potatoes NB (Grand Falls, NB). The genotypes ‘Russet Burbank’ (RB), ‘Atlantic’ (ALT) and ‘Yukon Gold’ (YG) were used. Thirty pounds (13.6 kg) of each genotype were obtained and subsequently stored in a cold room (4–10 °C) until use.

### 2.2. Cooking

Potato genotypes were individually processed and analyzed. For each genotype, the potatoes were washed and whole tubers were separated into four replicates, with ten tubers per replicate. For each replicate, potato tubers were cooked in boiling water until they reached acceptable softness, defined as when a stainless-steel knife could easily penetrate the tubers, which was validated with a meat thermometer indicating an internal temperature above 90 °C [23]. Upon cooking, the potatoes from each replicate pot were chopped using a standard kitchen knife into pieces less than 0.5 cm and cooled for 24 h at 4 °C.

### 2.3. Moisture Content

Before and after each drying treatment, the cooked tubers were weighed, which was calculated as previously described [23]. The calculated moisture content of freshly cooked samples was used to adjust starch measurements to dry weight. Adequate drying time for each treatment was determined in preliminary tests to ensure that samples were completely dried. The absence of any change in weight post-drying indicated that the samples were dried completely.

### 2.4. Drying Treatments

For each genotype and replicate, the cooked potato material was equally divided into five main treatments: fresh (control), FD, MVD, DDS and CHD. CHD was further subdivided into four treatments: CHD using a 82 °C water bath and infrared light (CHD1), CHD at 82 °C using an ultrasound water bath (CHD2), a combination of the two above CHD treatments (a 82 °C ultrasound water bath coupled with infrared light; CHD3), also known as ultrasound and infrared assisted conductive hydro-drying (UIACHD). Additionally, standard conductive hydro-drying using 95 °C water bath (CHD4) was tested. Between 10–20 g of cooked potato material was dried per treatment. A small fraction (~1–5 g) of each replicate and treatment was saved for microscopy. Material that was not used for immediate starch analysis was stored at −80 °C until analysis. For each drying treatment, samples were dried completely.

For the fresh treatment, starch analysis on cooked tubers was completed immediately after 24 h cooling to ensure complete retrogradation of starch. FD samples were completed at −50 to −60 °C and 0.85 mBar (0.64 mm Hg) (Gamma 1-16 LSC, Christ, Osterode am Harz, Germany) using previously established conditions [23]. After drying, samples were ground using a coffee grinder (CBG100SC, Black and Decker, Towson, MD, USA) and stored at −80 °C until starch digestibility analysis. Samples for MVD were shipped overnight to Enwave (Enwave Energy Corporation, Vancouver, BC, Canada) in an insulated plastic container with ice packs, and dried using the Enwave Microwave-vacuum dryer similar to References [35,36,37]. The microwave drying technology is comprised of a vacuum system, a microwave system, a sample chamber, as well as a ventilation/exhaust system. Inside the chamber, a container with the sample to be dried is placed and agitated during the drying process, while the proprietary microwave unit irradiates the sample material, dehydrating it. Adjusting the pressure within the chamber, which is controlled by the vacuum system, allows for increased dehydration in less time at a lower temperature compared to conventional microwave drying methods. Potato samples were dried using a microwave power of 2000 W for 15 min, followed by 1000 W for 55 min at 33 mBar. Afterwards, the chamber was vented and the access door opened to acquire the dried sample product. The DDS protocol was adapted from Godbout et al. (2016) [29]. The drying apparatus consisted of a pressure system and two electrovalves. The volume of the drying chamber was 0.34 L and was adapted from an oxygen pump (Parr-1108R, Moline, IL, USA). High-pressure was generated by a compressed air distribution network with 30% relative humidity. Gauges (Ashcroft, Stratford, CT, USA) measured pressure, both within and outside of the chamber. Low pressure was fixed as atmospheric pressure. The duration of each phase was regulated by the opening and closing of solenoid valves (Omega SV6003, Laval, QC, Canada). All components were connected with 2 inch perfluoroalkoxy tubes using plastic and stainless-steel Swagelok^®^ tubing fittings. The DDS drying consisted of 720 cycles of pressure variation per hour over 6 h, for a total of 4320 cycles. During each cycle, a primary (outer) valve was opened for 1 s to allow the pressure to increase within the chamber to 75 psi. Afterwards, the value was closed for 1 s and the samples slightly heated. The secondary (inner) valve was opened for 1 s, causing a pressure drop within the chamber and then closed for 2 s. The total drying cycle lasted 5 s and was completed at 27.5 °C. Once MVD and DDS drying was completed, the materials were sent back to McGill University and these were ground to a powder with a coffee grinder and stored at −80 °C until starch analysis.

Four different CHD setups of a batch laboratory scale ultrasound and/or infrared assisted conductive hydro-dryer were constructed according to References [34,38]. Setups for CHD treatments 1 to 3 consisted of a 28 kHz ultrasonic water bath (Beijing Ultrasonic, Beijing, China)) at 82.5 °C water temperature and 50% ultrasound power (166 W). A piece of Mylar sheet with 0.2 mm thickness was formed to fit over the water bath and an incandescent infrared lamp (Philips, Salina, KS, USA) with a reflector held over the dryer. The infrared lamp was connected to a dimer to adjust its power to 139 W. An electric fan with adjustable speed was used to provide lateral airflow (1 m/s) to remove vapors from the drying materials. A laboratory scale RWD [33] was also fabricated using a water bath (GCA Corporation 25AT-1, Precision Scientific Group, Chicago, IL, USA) for the CHD4 setup. The same type of Mylar sheet and the same airflow speed was used as previously described [33]. The water temperature was set to 95.0 ± 0.5 °C using a digital thermostat. All potato tuber samples for CHD treatments were dried for 5.5 min. Cooked and cooled potatoes for CHD drying were further cut into very small pieces (1–2 mm) and then spread onto the Mylar plastic membrane by rolling the potato material under a wax paper using a 50 mL Falcon tube to reach an approximate thickness of 1 mm. Dried samples were removed from the Mylar membrane, ground to a fine powder and stored at −80 °C until starch analysis. The membrane was washed with 70% ethanol between samples.

### 2.5. Starch Digestibility Assessment

Starch content (RS, DS and TS) was assessed using the Megazyme Resistant Starch assay kit (K-RSTAR) (Megazyme Int. Ireland Ltd., Wicklow, Ireland) as described previously [23]. The assay kit uses the methods developed Englyst et al. (1982; 1985; 1986; 1992) [39,40,41,42] but also further optimized by the works of Goni et al. (1996), Akerberg et al. (1998) and Champ et al. (1992) [43,44,45]. The application of the Megazyme Resistant Starch assay kit has been accepted by the AOAC International and AACC International Associations (AOAC Official Method 2002.02; AACC Method 32-40.0). Standard errors of ± 5% are expected for samples with more than 2% *w*/*w* RS.

In brief, samples (100 mg of the dried sample or 0.5 g of the fresh sample) were digested with 4.0 mL of pancreatic α-amylase containing dilute *Aspergillus niger* amyloglucosidase; AMG) for 16 h at 37 °C in a shaking water bath (Versa bath S 224, Waltham, MA, USA) at 200 strokes/min. Samples were then centrifuged at 1500× *g* for 10 min and the supernatant (DS portion) and pellet (RS portion) separated. DS samples were diluted to 100 mL with 100 mM sodium acetate buffer (pH 4.5). Aliquots of 0.1 mL with 10 µL of AMG (300 U/mL) were incubated for 20 min at 50 °C. Afterwards, 3.0 mL reagent enzyme mixture (glucose oxidase plus peroxidase and 4-aminoantipyrine; GOPOD), was added and further incubated for 20 min at 50 °C. D-Glucose content was determined by measuring the absorbance with a spectrophotometer (DU640, Beckman, CA, USA) at 510 nm. The buffer and GOPOD reagent were used as blank and D-glucose (1 mg/mL), as standards for starch content determination. The pellet (RS portion) was washed once with 99% *v/v* ethanol and twice with 50% *v/v* ethanol. For each wash, the samples were centrifuged at 1500× *g* for 10 min and the supernatant decanted. The pellet (RS portion) was resuspended using 2 M KOH buffer for 20 min in an ice water bath, on a magnetic stirrer. Exactly 0.1 mL of AMG (3300 U/mL) was added along with 8 mL of sodium acetate buffer (1.2 M) and 100 μL of AMG (3300 U/mL) and incubated for 30 min in a 50 °C water bath. After centrifugation, aliquots of 0.1 mL were treated with 3.0 mL of GOPOD reagent and incubated for 20 min at 50 °C. D-Glucose content was measured as described above for the DS portion. Each absorbance measurement was completed in duplicate. The glucose content of the collected supernatant and the digested pellet was calculated as per the kit instructions and summed (DS + RS) to calculate total starch (TS) content. Calculations included the conversion of absorbance to glucose content, weight and volume correction and a factor to convert the measured D-glucose content to anhydro-D-glucose that occurs in starch. For each genotype and treatments, 4 biological replicates were used (*n* = 4). All starch content was calculated on a dry mass basis in terms of a 100 g portion size (g/100 g DW), then calculated as % difference from the fresh control.

### 2.6. Fluorescent Microscopy

Fluorescent microscopy was used to visualize the surface structure of freshly cooked and cooled potato as well as the dried samples. In brief, each sample was transferred to glass slides with 2 drops of distilled water. To look at starch granule surfaces, a single drop of Calcofluor-white (1 g/L) (Sigma-Aldrich, Cat No. 18909) was added to the slide and allowed to react for 2 min. To view granule surfaces, the slides were viewed under dark field with an excitation wavelength of 365 nm. Image collection was performed at magnifications of 175× with a photomicroscope assembly (Leica EC3 camera mounted on a Leica DM2000 microscope with LASEZ (Leica Microsystems, Version 2.0.0, Buffalo Grove, IL, USA) imaging software).

### 2.7. Scanning Electron Microscopy (SM)

A Hitachi TM3000 (Hitachi High-Technologies, Tokyo, Japan) SM was used to visualize the surface structure of dried potato samples. A small layer of dried potato powder was mounted on a thin layer of carbon tape. Images were captured using TM3000 software (Hitachi High-Technologies, Version 02-03, Tokyo, Japan) using “Compo” and “Shadow1” image modes at 100×, 500× and 1000× magnification and obtained at 5 kV.

### 2.8. Statistical Analysis

Statistical analyses were completed using Jmp Pro 13.2.1 (SAS Institute Inc, Cary, NC, USA) and figures using Origin(Pro) (2018b, OriginLab Corporation, Northampton, MA, USA). Within each genotype, Dunnett’s test was used to compare drying treatments with the fresh treatment as the control. Outliers were determined using Grubbs’ test and excluded from the dataset if *p* < 0.05. Data was reported as the mean % difference between treatment and control ± standard error of the mean (SEM) and *p* < 0.05 was considered significant.

## 3. Results

### 3.1. Starch Profile

**Figure 1:**
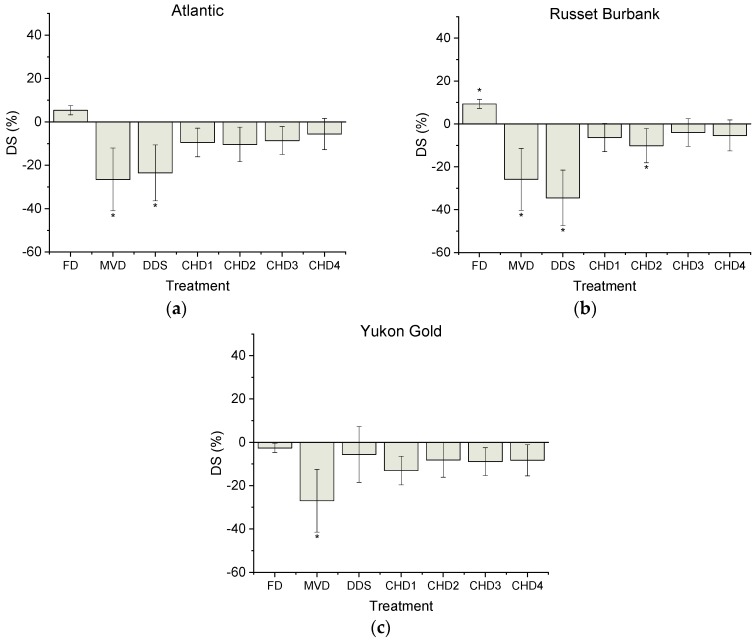
Digestible starch (DS) (percent difference) compared to fresh controls for Atlantic (ATL) (**a**), Russet Burbank (RB) (**b**) and Yukon Gold (YG) (**c**) potato cultivars. Baseline DS content of freshly cooked controls were 72.32 ± 3.05, 66.67 ± 0.920 and 69.98 ± 1.68 (g/100 g DW) for ALT, RB and YG respectively. Different drying treatments were assessed and compared to a fresh control using Dunnett’s test. Data is presented as mean ± SEM. For each genotype, * indicates statistically significant (*p* < 0.05) difference in comparison to control (fresh).

Genotype differences were observed in DS content after drying treatments. No statistical difference between the fresh control and FD were observed with ALT and YG, although for RB, DS was overestimated by 9.3 ± 2.1% with FD (*p* < 0.05) in comparison to fresh samples (Figure 1). DS values after MVD were significantly underestimated (*p* < 0.05) relative to controls by 26.5 ± 4.6, 25.9 ± 1.4% and 27.0 ± 2.6% for genotypes ALT, RB and YG respectively. DDS treatment was not significantly different from the fresh control in the genotype YG but DS content was significantly (*p* < 0.05) lower by 23.5 ± 7.0% and 34.5 ± 1.1% in ALT and RB, respectively. No significant changes in DS content were observed for all genotypes after CHD, except for CHD2 which led to an underestimation of DS (*p* < 0.05) in RB by 10.2 ± 1.5%. For all DS measurements, the observed differences were greater than the sensitivity of the kit used (see Supplementary Data, Figure S1).

**Figure 2:**
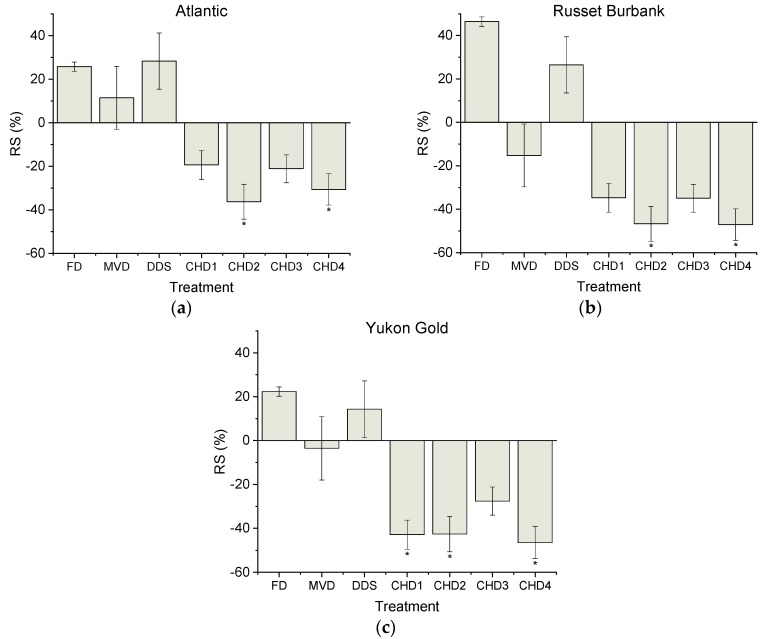
Resistant starch (percent difference) compared to fresh controls for Atlantic (**a**), Russet Burbank (**b**) and Yukon Gold (**c**) potato cultivars. Baseline RS content of freshly cooked controls were 6.20 ± 0.44, 5.98 ± 0.45 and 6.91 ± 0.23 (g/100 g DW) for ALT, RB and YG respectively. Different drying treatments were assessed and compared to a fresh control using Dunnett’s test. Data is presented as mean ± SEM. For each genotype, * indicates statistically significant (*p* < 0.05) difference in comparison to control (fresh).

Although not statistically significant, the RS content of ALT, RB and YG was overestimated (25.8 ± 2.1%, 46.4 ± 25.6% and 22.3 ± 20.0%, respectively) after FD (Figure 2). No significant differences in RS content were observed after MVD and DDS drying for any of the genotypes. CHD treatments were all associated with underestimated RS content, although the sensitivity to different CHD treatments varied with genotype. CHD1 (*p* < 0.05) was shown have underestimated RS content in YG by 42.9 ± 6.1%, whereas RS content was underestimated for every genotype with the CHD2 and CHD4 treatments. CHD3 showed no statistical difference between the fresh controls for ALT, RB and YG. For all RS measurements, the observed differences were greater than the sensitivity of the kit used (see Supplementary Data, Figure S2). The starch content that remained undigested (RS) for fresh and FD samples aligned with previously published literature [8,23,46].

**Figure 3:**
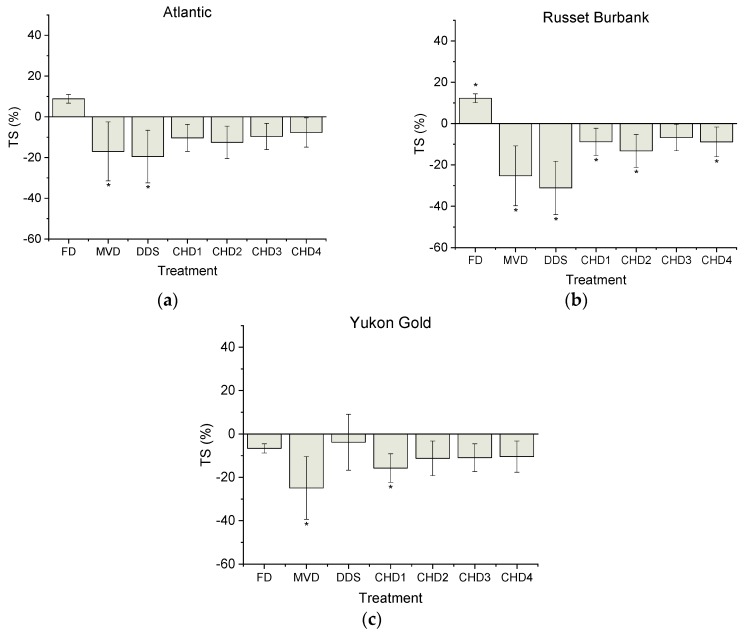
Total starch (percent difference) compared to fresh controls for Atlantic (**a**), Russet Burbank (**b**) and Yukon Gold (**c**) potato cultivars. Baseline TS content of freshly cooked controls were 78.52 ± 3.32, 72.65 ± 0.93 and 76.90 ± 1.90 (g/100 g DW) for ALT, RB and YG respectively. Different drying treatments were assessed and compared to a fresh control using Dunnett’s test. Data is presented as mean ± SEM. For each genotype, * indicates statistically significant (*p* < 0.05) difference in comparison to control (fresh).

The calculated percent differences in RS content between the fresh controls and drying treatments appeared to be the most variable, as compared to DS and TS (Figure 1, Figure 2 and Figure 3). For example, the greatest percent difference observed for RS was 47.0 ± 5.4% with CHD4 from RB, whereas the greatest deviation in observed DS content was 34.5 ± 1.0% with DDS from RB.

As seen with DS measurements, the effect of drying on TS content varied by genotype. No statistical difference between the fresh control and FD were observed with ALT and YG, although the TS content of RB was significantly (*p* < 0.05) overestimated by 12.3 ± 4.0% (Figure 3). Both MVD and DDS showed significant (*p* < 0.05) underestimation of TS content. Specially, the TS values after MVD were significantly underestimated (*p* < 0.05) for ALT, RB and YG relative to controls by 17.0 ± 8.3%, 25.3 ± 1.9%, 24.9 ± 3.3%, respectively. DDS treatment led to underestimated TS content in ALT and RB by 19.5 ± 7.1% and 31.1 ± 1.1%, respectively whereas no statistical difference was found in YG.

Based on the above starch profiles, TS, DS and RS content were significantly affected by all drying treatments in an unpredictable, cultivar-dependent manner. Similarly, previous work involving FD has also indicated that drying affects the digestibility of each cultivar in a different manner so that predictions could not made as to whether digestibility measurements would be either over- or under-estimated relative to control [23].

In the present study, assessment of RS in the genotypes appeared to be statistically unaffected by FD but still showed a major percent increase among the genotypes in RS content ranging from 22–46% relative to fresh controls. Likewise, no significant differences were observed in any of the starch measurements relative to controls for the CHD3 treatment for any of the genotypes; however, RS values among the three genotypes ranged from 21–35% lower than fresh samples.

### 3.2. Microscopic Observations

**Figure 4:**
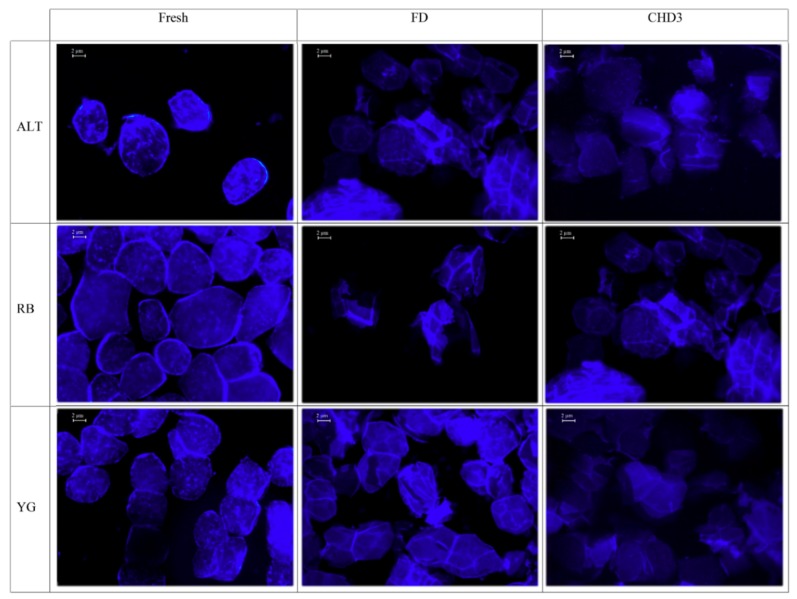
Fluorescent microscopy of three potato genotypes (rows) and three treatments (columns): fresh, freeze-dried (FD) and conductive hydro-drying (CHD3) at 175× magnification. For all cultivars, freshly processed potatoes show relatively intact starch granules with smooth walls (column 1) whereas FD (column 2) and CHD3 (column 3) showed granules with relatively less integrity and some cracking.

**Figure 5:**
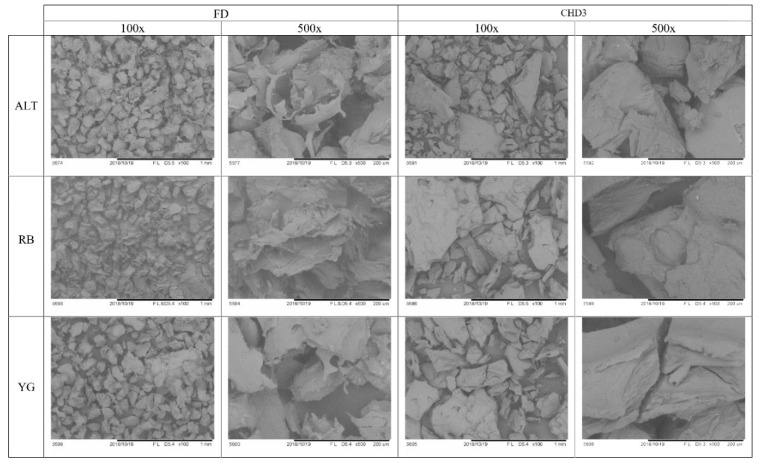
Scanning electron microscope (SM) images of three potato genotypes (rows) and two treatments (columns): freeze-dried (FD) and conductive hydro-drying (CHD3), at two magnifications (100× and 500×). For all cultivars, FD showed extensive cellular damage (columns 1 and 2), whereas CHD3 showed less granule damage although sheering can be observed (columns 3 and 4).

Freshly cooked potato samples demonstrate relatively intact swollen starch granules, which were uniformly stained with Calcofluor-white under fluorescent microscopy for all genotypes (Figure 4). Previous work has demonstrated that cooked potato starch granules remain intact although enlarged. The increase in starch granule size can be attributed to the swelling pressure that occurs during gelatinization of the starch within the cell during cooking [23,24]. No SM images were available for fresh samples since this technique requires the samples to be dried for imaging. FD was also investigated using microscopy, due to the common use of this method in industry prior to nutritional assessments. As observed previously, FD caused cracks and fragmentation of the starch granule cell wall integrity [23,24], which was observed under both fluorescent microscopy and SM (Figure 4 and Figure 5). FD can alter the starch granule integrity and so lead to an increased permeability of potato starch to enzymatic digestion. Similar structural damage induced by drying of raw potatoes has been associated with increased starch digestibility [21,47]. Samples dried by CHD3 were also assessed by fluorescent microscopy and SM since no significant differences were observed in any of the starch measurements relative to controls for any of the genotypes. The starch granule surface of potatoes after CHD3 was more intact for all genotypes as compared to FD, although some cellular damage to structure integrity and shearing was observed for all genotypes (Figure 4 and Figure 5). Although RS content was underestimated after CHD3 treatment, this drying method has been reported as a promising alternative to drying heat sensitive samples and could provide a less costly alternative to FD [34].

## 4. Discussion

All starch quality content (TS, DS and RS) measurements were affected by drying treatments in a genotypic-dependent manner. This finding is mostly likely due to inherent differences in starch structure and content that can vary between genotypes [23,25,26], which could lead to their variable responses to the drying treatments [23]. Although samples were dried to completion and compared on a dry weight basis, minor differences in end-point moisture content could contribute to the observed differences in starch content and vary by genotype. The results showed that the DDS and MVD treatments are not appropriate drying tools for the investigation of starch profiles of cooked potatoes since major differences in starch profiles were observed relative to fresh samples. FD is a common drying method used for quantification of TS, DS and RS content of potatoes [2,8,22,46,48,49]. In the present study, genotype-dependent variations in starch profiles were observed with FD. Although the effect of FD on the estimation of DS and TS content of ALT and YG was not statistically different relative to fresh controls, RB that was FD showed significantly greater DS and TS content relative to fresh controls. These results are in agreement with previous work indicating that FD is not a reliable tool for screening starch profiles among potato genotypes [23]. Previous results have shown that FD can mechanically damage the starch granule cells of potato starch and so alter the permeability of starch grains to enzymatic digestion [21,47]. The above phenomena could be genotype dependent, most likely due to the inherent differences in starch characteristics among varieties. Protein aggregation could explain the measured differences in starch profiles as starch-protein complexes can interfere with starch digestibility [50,51,52]. It is conceivable that the disruption of starch granules as shown by microscopy for the various drying treatments could have enhanced formation of the above complexes and led to the distorted measurement of TS versus fresh values (Figure 3).

All the CHD treatment combinations showed genotype-dependent differences in RS, DS and TS. CHD3, otherwise known as UIACHD, showed no statistical differences in DS, RS and TS compared to control. Although not significant, the percent difference in RS using CHD3 was still high (34.9 ± 7.7%). Future studies, however, could consider the use of UIACHD towards drying of cooked potatoes for high-throughput screening of DS or TS content as these measures showed minimal variations relative to the fresh controls. Microscopic analysis showed that the structural integrity of potato samples after UIACHD was significantly less disturbed than FD, although both methods showed differences in comparison to fresh samples. UIACHD can be considered as a possible alternative to FD as UIACHD is faster, more energy efficient and less costly [33,34]. Due to the association of RS with decreased risk of chronic diseases [4,8,20], starch assessments have recently focused to select potato genotypes with the greatest RS content. RS content, however, generally showed the greatest variability following the drying treatments and so caution is needed when interpreting results obtained from dried potato samples for RS analysis.

After investigation of multiple non-conventional drying methods, it is apparent that for accuracy of starch profile measurements, use of freshly cooked samples is still important, particularly with respect to RS content. The UIACHD method, however, shows promise as a drying process towards accurate evaluation of the DS and TS content of cooked potatoes and so this technology could be further investigated in that regard. Screening genotypes for optimal starch profiles using fresh cooked potato samples is a difficult process due to the seasonal demands of harvested food crops, particularly since storage time/conditions are confounding variables that affect the nutritional content of potatoes. Hence, identification of other drying alternatives for high-throughput RS analysis is a key next step to support the commendable initiatives by potato breeders to identify table stock with improved starch profiles for consumers.

## Supplementary Materials

The following are available online at https://www.mdpi.com/2304-8158/8/9/382/s1, Figure S1: Absorbance values of DS for Atlantic (S1a), Russet Burbank (S1b) and Yukon Gold (S1c) potato cultivars. Data is presented as mean ± SEM, Figure. S2: Absorbance values of RS for Atlantic (S2a), Russet Burbank (S2b) and Yukon Gold (S2c) potato cultivars. Data is presented as mean ± SEM.
