# Supplementary figures and images for: Effect of Non-Conventional Drying Methods on In Vitro Starch Digestibility Assessment of Cooked Potato Genotypes
Source: Foods. 2019 Sep 2;8(9):382. doi: 10.3390/foods8090382 (PMC6770100; doi:10.3390/foods8090382)

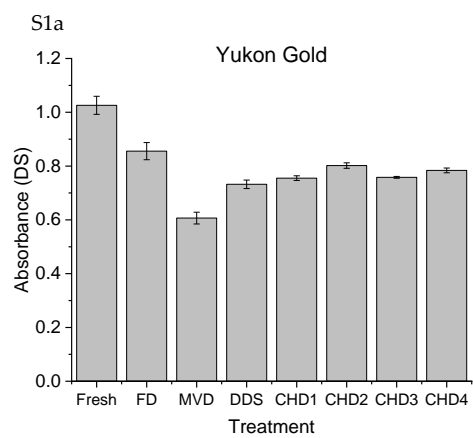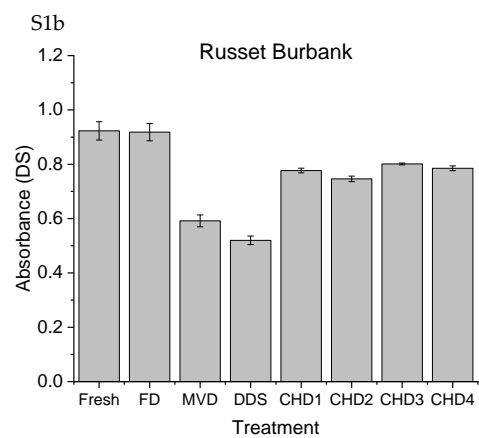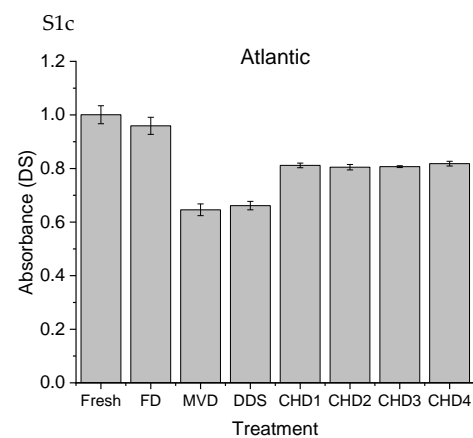

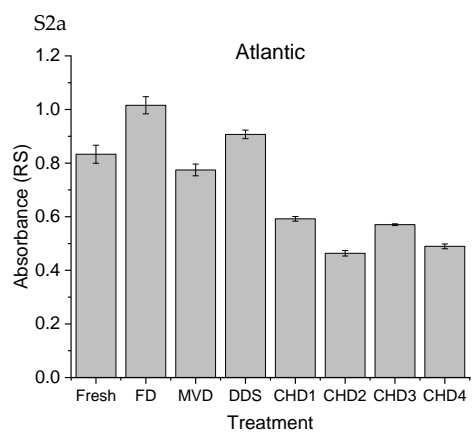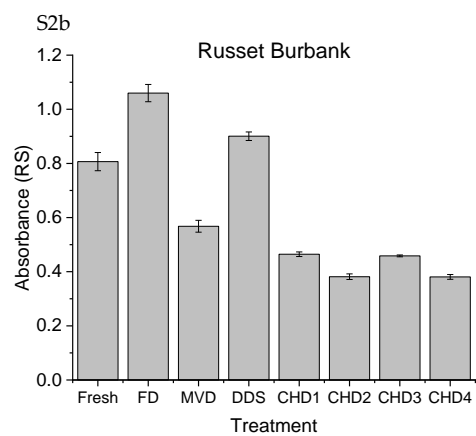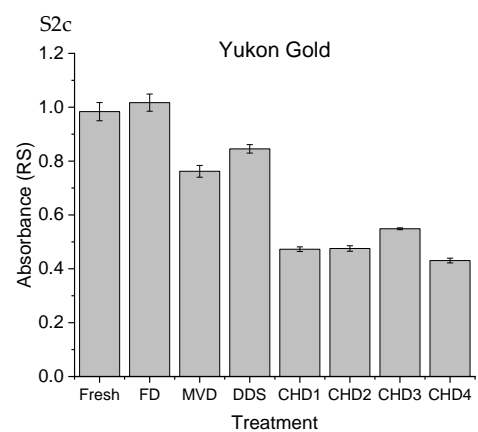

Supplement: Supplementary file 1 [file foods-08-00382-s001.pdf]
